# Preparation and Properties of SBS-g-GOs-Modified Asphalt Based on a Thiol-ene Click Reaction in a Bituminous Environment

**DOI:** 10.3390/polym10111264

**Published:** 2018-11-13

**Authors:** Jing Li, Meizhao Han, Yaseen Muhammad, Yu Liu, Zhibin Su, Jing Yang, Song Yang, Shaochan Duan

**Affiliations:** 1 Guangxi Key Laboratory of Petrochemical Resource Processing and Process Intensification Technology, School of Chemistry and Chemical Engineering, Guangxi University, Nanning 530004, China; lijingsophia1234@163.com (J.L.); myyousafzai@gmail.com (Y.M.); 2 Guangxi Colleges and Universities Key Laboratory of New Technology and Application in Resource Chemical Engineering, School of Chemistry and Chemical Engineering, Guangxi University, Nanning 530004, China; hmzsourire@163.com (M.H.); gxusuzhibin@163.com (Z.S.); Betsy0828@163.com (J.Y.); qq314593113@163.com (S.Y.); duanshaochan@163.com (S.D.); 3 Insitute of Chemical Sciences, University of Peshawar, Peshawar 25120 KP, Pakistan; 4 Guangxi communication investment Technology Co. Ltd., Nanning 530004, China

**Keywords:** thiol-ene click reaction, asphalt environment, GOs-SH, SBS-g-GOs-modified asphalt, textural characterization, mechanical properties

## Abstract

Styrene-butadiene styrene graphene oxide nanoplatelets (SBS-g-GOs)-modified asphalt was prepared by reacting thiolated GOs (GOs-SH) with SBS in asphalt using a thiol-ene click reaction. The temperature resistance and mechanical properties of asphalts were analyzed by dynamic shear rheology (DSR) and multiple-stress creep-recovery (MSCR) tests, which revealed that an optimum amount of GOs-SH (0.02%) can effectively improve the low temperature and anti-rutting performance of SBS asphalt. Segregation experiments showed that SBS-g-GOs possessed good stability and dispersion in base asphalt. Fluorescence microscopy results revealed that the addition of GOs-SH promoted the formation of SBS network structure. Textural and morphological characterization of GOs-SH and SBS were achieved by Fourier transform infra-red (FT-IR) spectroscopy, energy-dispersive X-ray spectroscopy (EDX), atomic-force microscopy (AFM), X-ray diffraction (XRD), and scanning electron microscopy (SEM), while surface chemical composition was tested by X-ray photoelectron spectroscopy (XPS). Based on textural characterization data, a suitable reaction mechanism was proposed that involved the preferential reaction between GOs-SH and 1,2 C=C of SBS. The currently designed GOs-SH incorporated asphalt via thiol-ene click reaction provides new ideas for the preparation of modified asphalt with enhanced mechanical properties for target-oriented applications.

## 1. Introduction

The performance of ordinary asphalt limits the heavy traffic load-capacity and service life [1,2,3,4]. For better performance, various modifiers are applied for the preparation of modified asphalt. Among these, styrene-butadiene-styrene (SBS) block copolymer-modified asphalt is widely used in highway paving due to its better road performance [5,6,7]. However, due to issues like poor thermal stability during storage, improving the performance of SBS-modified asphalt has attracted greater attention of many research groups [8,9,10]. For instance, Wu et al. [11] found that 4-aminobenzophenone grafted onto SBS by free radical polymerization can effectively improve the elastic performance of pristine SBS. Cong et al. [12] used benzoyl peroxide as an initiator to graft maleic anhydride onto SBS, along with enhanced high temperature performance and the storage stability of the modified asphalt. Chemically, the polybutadiene segment of SBS contains a large number of C=C bonds, which can be divided into three structures: 1,2 C=C, and cis and trans 1,4 C=C. When the grafting replaces ortho-α-H of C=C [13,14], the product still contains a large number of double bonds, especially 1,2 C=C, which is easily oxidized and hence can in turn cause SBS aging. On the contrary, when the reactants are excessively grafted onto 1,4 C=C structure, the resulting SBS product greatly loses its elasticity [15,16,17]. Studies of Schapman et al. [18] and Lotti et al. [19] have shown that 1,2 C=C activity is the highest in SBS among all C=C species. Li et al. [20] found that the thiol group (–SH) in mercaptoethanol can be preferentially reacted with 1,2 C=C in SBS by a thiol-ene click reaction. They found marked improvements in segregation and thermal storage properties of SBS-g-OH in asphalt, and mild improvements in mechanical properties were observed as well.

Click chemistry as an emerging synthetic concept has been widely applied by many researchers in various fields accredited to its simplicity, high efficiency, and wide range of applications [21,22,23,24,25,26,27]. Fouassier [28] pointed out prospective applications of thiol-ene click reaction in the field of photocurable adhesives and coatings. Hoyle et al. [29] summarized the research status of thiol-ene photochemical reactions concluding the broad prospects of thiol-ene click reaction in the preparation of polymer materials with adjustable physical and mechanical properties. Hawker’s [30,31] and Wooley’s [32,33,34] groups have done a lot of in-depth research on the application of thiol-ene reactions.

In terms of mechanical properties, graphene nanoplatelets (GNPs) have been widely studied for their excellent performance [35,36,37]. Among these, the high mechanical properties of GNPs greatly favor their application in the preparation of modified asphalt. When GNPs are added to asphalt, they fill the asphalt system through their uniform dispersion, thereby increasing the overall mechanical strength of the modified asphalt. This effect is analogous to that of a nanomaterial modifier (for example, carbon nanotubes [38]). However, the cost of GNPs decreases significantly as advancements occur in production technology. Gao et al. [39] found that adding GNPs can improve road-surface rutting performance at an intermediate temperature, but compaction at a lower temperature has an adverse effect on rutting performance. Le et al. [40] found that GNPs as a modifier and composited with SBS to prepare modified asphalt have serious compatibility problems. In our previous studies [41,42,43], polystyrene and octadecyl amine grafting onto GNPs showed the enhancing effect on the mechanical properties of SBS-based modified asphalt. However, in these studies, GNPs and SBS were assumed to have been modified physically rather than chemically. Interestingly, the surface of the oxidized GOs contains a large number of oxidized groups, which facilitates its better modification, extending the range of GNPs properties.

In the current study, the SBS network structure with GOs was formed as a junction via the thiolation of GOs (GOs-SH) and by relying on the preferential reaction of 1,2 C=C on SBS via the thiol-ene click reaction. In order to avoid the self-encapsulation of GOs-SH with multiple SBS chains (due to the presence of multiple sulfhydryl groups), they were simultaneously added to the asphalt. Furthermore, the shearing effect in shearing also helped to reduce the self-wrapping phenomenon of SBS during SBS-g-GOs-modified asphalt preparation. By measuring two indicators (dynamic shear rheology (DSR) test and multi stress creep recovery (MSCR) tests), the modification properties of SBS-g-GOs were compared with SBS-modified and GNPs/SBS-modified asphalts. GOs-SH were microscopically characterized by Fourier transform infra-red spectroscopy (FT-IR), energy-dispersive X-ray spectroscopy (EDX), atomic-force microscopy (AFM), X-ray diffraction (XRD), and scanning electron microscopy (SEM), while the species taking part in the thiol-ene click reaction between GOs-SH and SBS were explicated by X-ray photoelectron spectroscopy (XPS).

## 2. Preparation of SBS-g-GOs-Modified Asphalt

### 2.1. Materials 

All the materials used were of analytical grade and used without further purification. Base asphalt (SK-70A) was supplied by Xiamen Walter Group Co., Ltd., Xiamen, China. SBS block Polymer (SBS, T6302H) was supplied by Dushanzi petrochemical branch, China national petroleum Co., Ltd. (Karamay, China). Sublimation sulfur (CP) was provided by Sinopharm group chemical reagent Co., Ltd. (Shanghai, China). Multilayer GO nanoplatelets (film diameter: 10–50 μm, thickness: <20 nm, specific surface area: 100–300 m^2^/g) were purchased from Suzhou carbon abundance electronic technology Co., Ltd., Suzhou, China. Silane coupling agent 3-Mercaptopropyltriethoxysilane (KH580) was supplied by Aladdin industrial corporation, Shanghai, China. Absolute ethyl alcohol and ammonium persulphate were purchased from CP, Guangdong chemical reagent engineering technology research and development center, Shantou, China. 

### 2.2. Preparation Method

#### 2.2.1. Preparation of GOs-SH

**Figure 1:**
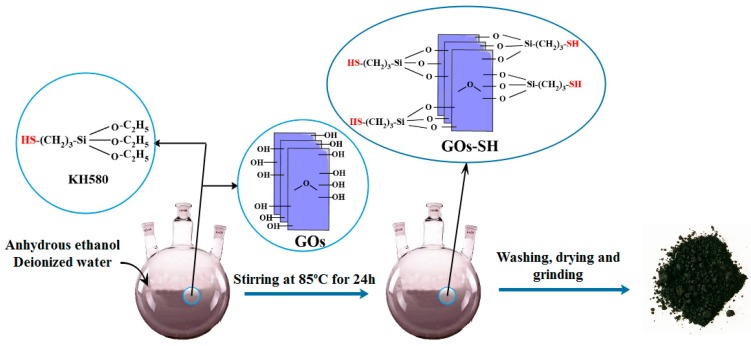
Schematic representation of synthesis process of GOs-SH.

Figure 1 shows the synthesis process of GOs-SH. 0.5 g of GOs and 50 mL absolute ethanol was separately added into 250 mL three-necked flask. After ultrasonic dispersion for 20 min, 25 mL deionized water and 1.25 mL of KH580 were added and evenly shaken at 85 °C in water bath for 24 h. Upon the completion of reaction, the product was washed with absolute ethanol three times. The product was vacuum dried at 80 °C, and a black flaky solid (GOs-SH) was obtained, which was ground to powder. During the reaction, the ethoxy groups of 3-Mercaptopropyltriethoxysilane (KH580) undergo hydrolysis to form highly reactive silanol groups. These silanols undergo hydrogen bonding among each other in the solution to form a network and then form covalent bonds with GO surface (by the elimination of water molecule). The GOs contain multiple layers with variable number of –OH groups. At the same time, each KH580 molecule contains three –C_2_H_5_ groups. Therefore, during the grafting reaction, the same KH580 molecule may be grafted on the same or on different sheets. With this grafting method, different layers of GOs can be interconnected by KH580 molecules that form elastomer structure, as shown in Figure S1.

**Figure 2:**
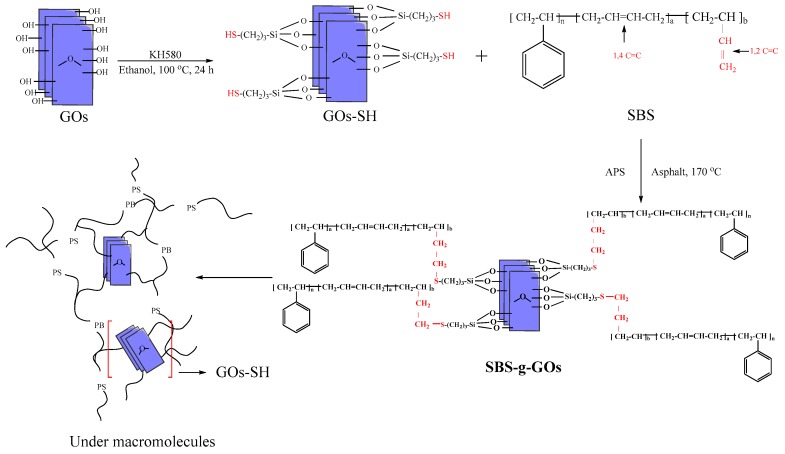
Proposed mechanism of thiol-ene click reaction of SBS-g-GOs.

After the addition of GOs-SH, the thiol-ene click reaction between SBS and GOs-SH was induced by APS as initiator. Credited to the high activity of 1,2 C=C center in SBS, GOs-SH preferentially attacks this section. Furthermore, as there are multiple –SH in a single GOs, the same GOs can react with multiple 1,2 C=C to connect different SBS molecules (same or different SBS chain) to each other. So, the entire reaction process is transferred to the asphalt environment. The thiol-ene click reaction would occur with the large particles of SBS that have been shear-dispersed. According to this method, GOs with excellent mechanical properties become the connection points in the SBS network. The entire reaction process and proposed mechanism is shown in Figure 2.

In a previous study, it was found that if SBS is dissolved and thiol-ene reaction is carried out with GOs-SH, the product shows uneven distribution of GOs resulting in two extremes of the performance, i.e., the performance is good in areas with a large distribution of GOs, while it is degraded in areas having lesser GOs. This was mainly attributed to the linear structure of SBS, which on stirring after dissolution allows GOs-SH to easily undergo a thiol-ene reaction on the same SBS molecular chain. This causes GOs-SH to lose the effect of linking multiple SBS molecular chains, as shown in Figure 2. In order to tackle this scenario, GOs-SH and SBS were added directly to the asphalt and, the modification of the asphalt is simultaneously carried out during the reaction. It was found in a previous study focusing on the modification mechanism of SBS in asphalt [44] that SBS does not dissolve in the asphalt but exists in the form of particles under the action of a high-speed shear. SBS swells as it absorbs light components in the asphalt, thus unfolding its original chain structure. This change in SBS in asphalt creates the possibility of GOs-SH connecting multiple SBS molecular chains. The small granular SBS during the initial process makes it difficult for all thiol groups (–SH) on GOs-SH to completely react with the same SBS chain. With the development of the SBS chain, the opportunity for the GOs-SH grafted on it to react with other SBS chains increases.

#### 2.2.2. Preparation of SBS-g-GOs-modified asphalt

The production procedure for modified asphalt followed by Guangdong Traffic Testing Center and Guangxi Trading Technology Co., Ltd., Guangdong, China is displayed in Figure S2 [41,45]. In this study, the ratio of base asphalt, SBS, and stabilizer (sublimation sulfur) is 100:5:0.15, while GOs-SH and APS were added to SBS at the beginning of shearing during SBS-g-GOs-modified asphalt preparation. 

### 2.3. Performance and Characterization Analysis

Ductility, softening point, and penetration were tested to preliminarily judge the modification effect of GOs-SH. DSR and MSCR (Bohlin CVO) test were used to determine the viscoelastic and anti-rutting properties of SBS-g-GOs-modified asphalt, respectively. Segregation experiments were used to determine the stability of SBS-g-GOs in asphalt. FM (IMAGER.Z2, Carl Zeiss, Oberkochen, Germany) was used to observe the presence and distribution of newly formed SBS-g-GOs in asphalt after the addition of GOs-SH.

In microscopic characterization, FT-IR (iS50, US NICOLET Co., Ltd., Madison, WI, USA, and potassium bromide as a background) and EDX (SU8220, Hitachi, Tokyo, Japan, accelerating voltage: 5KV) were applied to get insight about the composition of GOs-SH [46]. XRD (SMARTLAB3KW, Rigaku, Tokyo, Japan) was used to analyze the change in structure before and after GOs modification. SEM (SU5000 and SU8220, Hitachi, Tokyo, Japan accelerating voltage: 10KV and 5KV) and AFM (Dimension FastScan, BRUKER, Tap mode, Karlsruhe, Germany) were used to analyze the change in appearance and mechanical properties of the pre and post GOs-modified samples, respectively. The Sputter Coaster (MSP-2S, IXRF, Houston, TX, USA, 8–10 pa of vacuum degree, 60 s of vacuum time, 15 s of coating time, discharge current: 38 mA) was used to spray gold film (about 7.5 nm thickness) on SEM samples. XPS (ESCALAB 250XI+, Thermo, Waltham, MA, USA) was applied to characterize the surface species electronic states of SBS-g-GOs in asphalt.

## 3. Results and Discussion

### 3.1. Three Indicators

**Figure 3:**
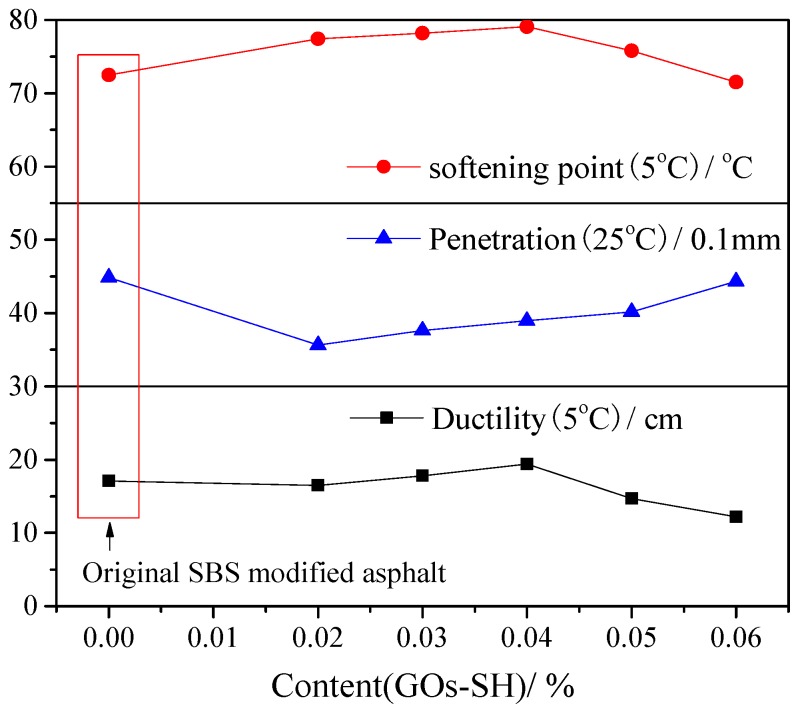
Tests results of modified asphalt with different GOs-SH content.

The modification effect of GOs-SH in terms of three indicators, i.e., penetration, softening point, and ductility were determined in accordance with JTG E20–2011 [47] and the results are shown in Figure 3.

Figure 3 suggests that increasing GOs-SH content improves the ductility and softening point initially, followed by a decrease. 0.04% GOs-SH was observed with best modification effect, i.e., increasing softening point and ductility by about 9.14% and 12.35%, respectively, compared with original SBS-modified asphalt. On the contrary, penetration observed a decrease by 0.02% GOs-SH followed by a continual increase. These results could be attributed to the fact that an appropriate amount of GOs-SH (0.02–0.04%) preferentially reacts with 1,2 C=C in SBS, thereby improving the performance of SBS. Further addition of GOs-SH (0.06%) can react with 1,4 C=C after the complete consumption of 1,2 C=C in SBS, thereby reducing the rubber properties of SBS itself. Thus, from Figure 3, one can conclude that 0.02% GOs-SH can effectively improve the plasticity, ductility, and visco-elastic properties of SBS-modified asphalt.

### 3.2. DSR Test

DSR is considered a highly accurate and efficient instrument in determining the viscoelastic properties of asphalt [48]. In frequency (0.1–10 Hz) and temperature (46–82 °C)-scanning mode, the complex modulus (G*) and the phase angle (*δ*) of asphalt can be determined by controlling strain or stress. Based on the value of G* and *δ*, other physical quantities representing asphalt viscoelastic energy such as loss factor (tan*δ*), storage modulus (*G*′), and loss modulus (*G*′′) can be calculated by Equations (1) and (2), respectively.

(1) $$G^{\prime }=\left|G^{*}\right|\cos\delta \;$$

(2) $$G^{"}=\left|G^{*}\right|\sin\delta \;$$

In our previous study, 0.05% GNPs contents were found with optimized modification effect in SBS asphalt [41]. In order to reflect the difference in the effect of GOs-SH and GNPs on asphalt modification, 0.05% GNPs/SBS-modified asphalt was prepared as a control sample for comparison.

#### 3.2.1. Variation of *G*′ with GOs-SH Content

**Figure 4:**
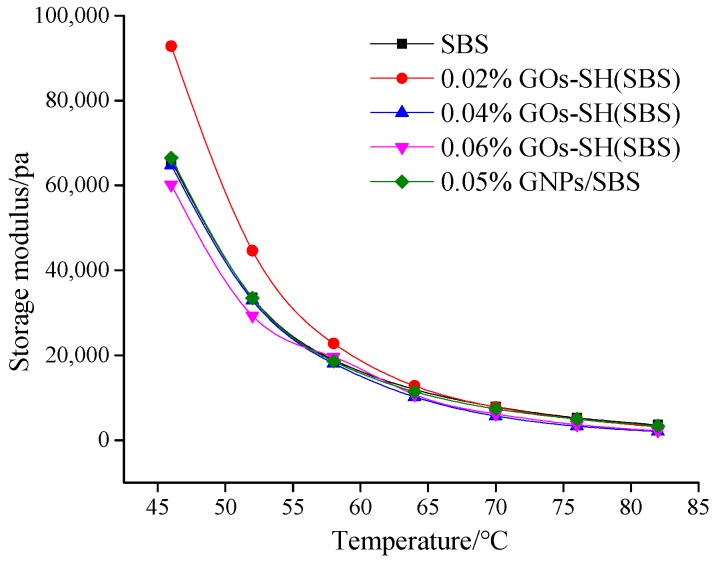
Effect of GOs-SH content on G’ as a function of temperature at 10 Hz.

The value of *G*′ proportionally represents the elastic properties of asphalt. It can be seen in Figure 4 that at 10 Hz, with increasing temperature and GOs content, *G*′ of each group of asphalt decreases. Compared to original SBS asphalt, 0.02% GOs-SH modified asphalt exhibited the largest (40.86% at 46 °C) improvement, while 0.06% GOs-SH improved the least. Furthermore, the modification effect of 0.02% GOs-SH was better than 0.05% GNPs reported in previous study [41].

**Figure 5:**
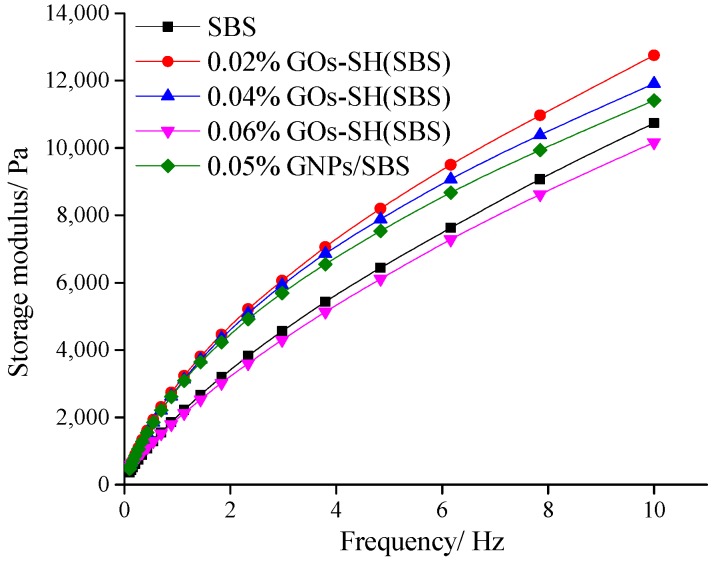
Effect of GOs-SH content on *G*′ as a function of shear frequency at 64 °C.

Similarly, in Figure 5, 0.02% GOs-SH was the largest (18.73%), while 0.06% GOs-SH had the least effect compared to original SBS asphalt. With increasing frequency, the *G*′ of each group increased, while with increasing GOs content, it drastically decreased. These results suggested that adding proper amount of GOs-SH can effectively improve the elastic properties of asphalt. 

#### 3.2.2. Effect of GOs-SH Content on tan*δ* and *G*′′

**Figure 6:**
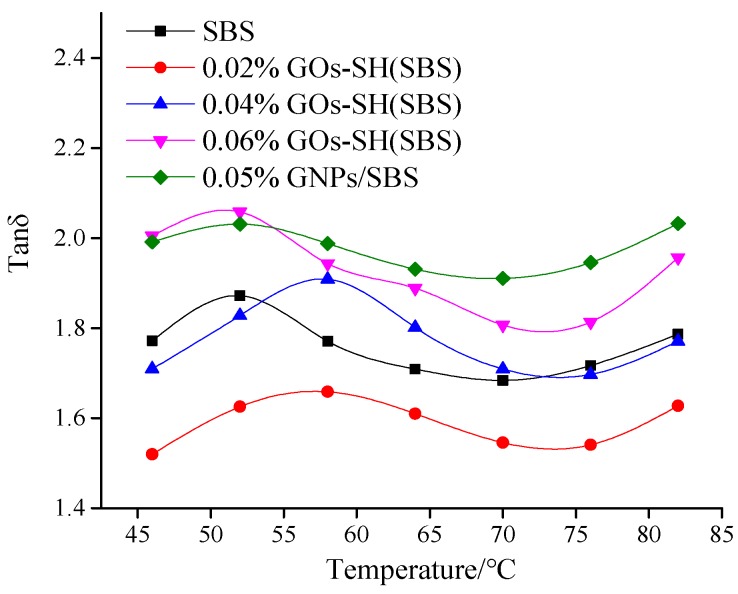
Variation in Tan*δ* (10 Hz) with temperature with variable GOs-SH content.

**Figure 7:**
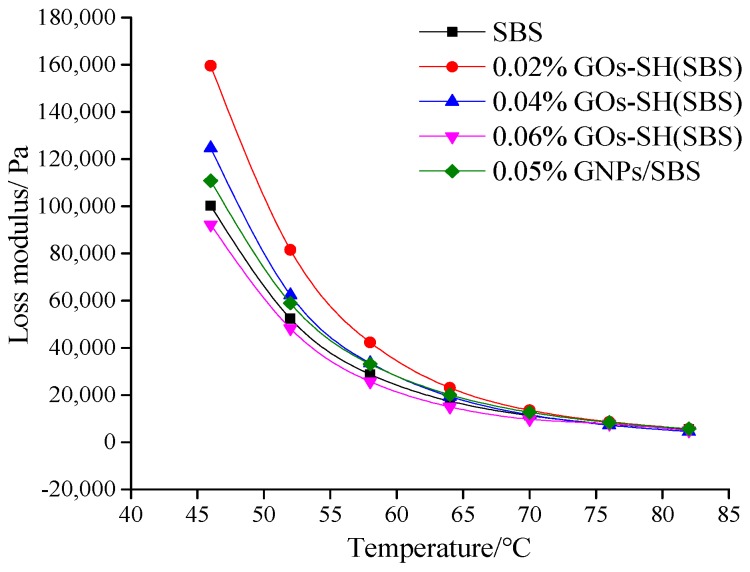
Variation in loss modulus *G*′′ (10 Hz) with temperature at variable GOs-SH content.

The value of tan*δ* and *G*′′ represent the visco-elastic properties of asphalt. Figure 6 shows an increase in tan*δ* with increasing temperature and GOs content at 10 Hz. It was found that 0.02% GOs-SH possessed the highest tan*δ* with an improvement of 9.04% compared to original asphalt at 46 °C, while that of SBS was the lowest. These data once again suggested that an optimum amount of GOs-SH can effectively improve the elasticity of asphalt, while 0.02% GOs-SH was again proved to have better effect than 0.05% GNPs. Similarly, Figure 7 suggests that with rise in temperature and GOs contents, *G*′′ of each group of asphalt decreases. 0.02% GOs-SH added asphalt was once again found with maximum improving effect, i.e., increasing *G*′′ by 59.16% at 46 °C compared to base asphalt, while 0.06% GOs-SH showed the mildest effect. 

The test results of *G*′, tan*δ,* and *G*′′ could be due to the fact that GOs-SH having an elastomer structure is grafted in the SBS network after the thiol-ene click reaction, thereby further improving the elastic properties of the resulting composite network. However, an amount of GOs-SH exceeding that required for the complete reaction of 1,2 C=C in the SBS network reacts with 1,4 C=C, thereby reducing the elasticity of SBS itself, which is visible by mild effect of 0.06% GOs-SH content.

#### 3.2.3. Determination of the High-Temperature Asphalt Grade (PG Grade)

By calculating the rutting factor (RF) G*/sin*δ*, the high-temperature grade (PG) of each group of asphalt is classified in accordance with standard classification [49,50]. The calculation results of RF for each group are shown in Table 1.

According to the asphalt high-temperature grade-classification requirements, the RF value of each unaged asphalt is greater than 1 kPa [51,52]. However, after the rotating film oven treatment (RTFOT) short-term aging, RF value of 0.06% GOs-SH/SBS-modified asphalt was 1.97 kPa, indicating that the high temperature grade of this group of asphalt is reduced to PG76. This is again attributed to the fact that a suitable amount of GOs-SH is required for grafting to 1,2 C=C on SBS, to prevent SBS from aging and relying on the excellent mechanical properties of GOs-SH to increase the strength of the composite network. However, excessive GOs-SH will be grafted to 1,4 C=C after saturation at 1,2 C=C, which ultimately reduces the rubber properties of SBS itself. This results in decreased strength of the composite network, leading to lower rating of the high-temperature performance of asphalt than original SBS.

### 3.3. MSCR Test

Viscous-elastic materials undergo creep upon exposure to stress, and once the stress is removed, some part of creep deformation is recovered, while the irrecoverable partial deformations are added to the next load cycle. The road surface is subjected to such repeated loading and unloading by the vehicle, and hence leads to the accumulation of such deformation. The MSCR test simulates this strain accumulation process, which reflects the cumulative deformation of the asphalt material under repeated one-way loading and unloading conditions, which is assumed to be independent of the deformation characteristics of asphalt. MSCR test (AASHTO’s test protocol TP70-10) has been effectively applied to measure the anti-rutting performance of asphalt and hence was applied to test SBS-g-GOs-modified asphalt in this study [49]. According to the test results mentioned in Section 3.2.3 high-temperature asphalt grade, asphalt samples with variable amount of GOs-SH were tested at 82 °C and compared with pristine SBS. As PG grade of 0.06% GOs-SH(SBS) has reduced to PG76, it was not included in this comparison.

**Figure 8:**
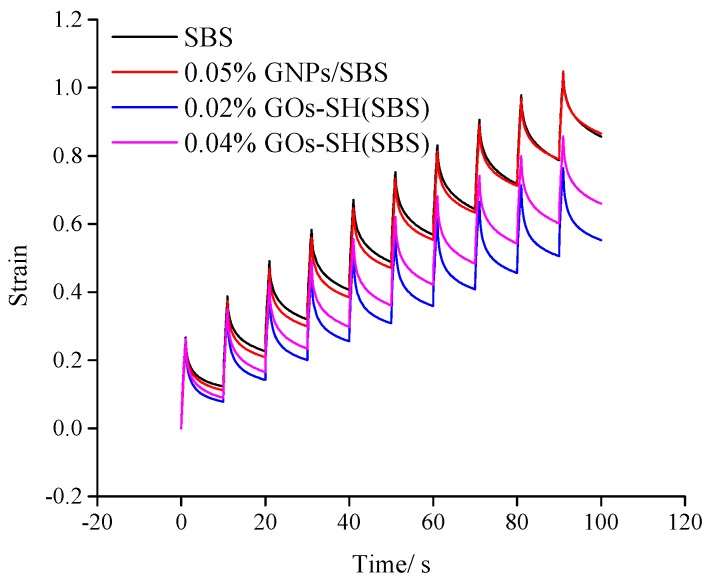
Time-strain relation of different asphalt samples at 0.1 KPa.

**Figure 9:**
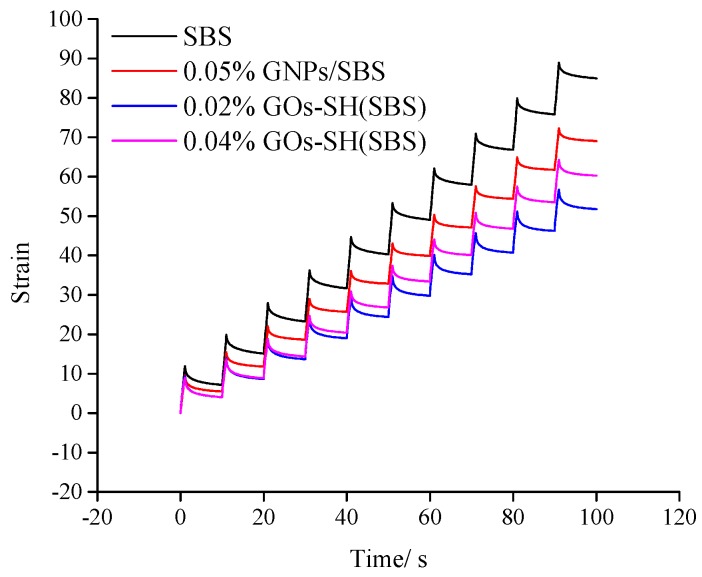
Time-strain relation of different asphalt samples at 3.2 KPa.

Figure 8 and Figure 9 show the creep performance of each group of asphalt under two stresses (100 Pa and 3200 Pa) within 10 cycles of the instrument. Each cycle includes a one-second loading and a nine-second unloading process, which represent the rising and falling parts of the staircase curve, respectively. The highest and lowest point of the ascending and descending phase represent the peak strain (γ_p_) and residual strain (γ_nr_) at the end of single-cycle loading and unloading process, respectively, and the strain at *T* = 0 is termed as initial strain (γ_0_). A lower slope of each line indicates stronger ability of asphalt to return to its original shape under external forces. Obviously, it can be seen that the strain trend of 0.02% GOs-SH(SBS) under both stresses is the lowest, suggesting its strongest recovery after deformation under external load, which is indicative of its improved resistance to external forces. Through the time-strain curves of each sample obtained from MSCR experiment, the response rate (R) and the creep compliance (J_nr_) representing the anti-rutting performance of the asphalt under different stresses can be calculated according to Equations 3 and 4, respectively.

R = (γ_p_ − γ_nr_)/(γ_p_ − γ_0_) (3)

J_nr_ = (γ_nr_ − γ_0_)/τ (4)

A higher R value in Table 2 indicates that the bitumen has higher elasticity and ability to return to the original state after being subjected to the same stress. It can be seen that under both high (3.2 kPa) and low (0.1 kPa) stress, R of 0.02% GOs-SH(SBS) (R_0.1_ = 0.745 and R_3.2_ = 0.494) were the highest mounting to an improvement of about 16.44% and 44.87%, respectively, compared with SBS group (R_0.1_ = 0.675 and R_3.2_ = 0.341). Similarly, 0.04% GOs-SH(SBS) showed an improvement in R of 10.37% and 22.87%, respectively, at two test stresses. Higher value of R for GOs-SH(SBS) suggested the improved elasticity of bitumen by the addition of suitable amount of GOs-SH.

The value of J_nr_ represents the irreversible creep of asphalt after 10 test cycles and a fixed test-stress in which a higher value shows lesser degree of recovery by the bitumen. Table 2 shows that under both stresses, the J_nr_ values of 0.02% GOs-SH(SBS) (J_nr0.1_ = 0.553 and J_nr3.2_ = 1.618) were the lowest, showing a reduction of about 35.38% and 39.06%, respectively, compared with the SBS and 0.04% GOs-SH(SBS)-modified asphalts. J_nr_ data concluded that GOs-SH addition considerably enhances the ability of bitumen to resist permanent deformation caused by external forces. 

These results for R and J collectively showed that proper amount of GOs-SH addition can improve the anti-rutting performance of asphalt, which decreases with increasing GOs content. Beyond certain level, GOs-SH is grafted to 1,4 C=C on SBS, which destroys the original rubber properties of SBS. This also reduces the strength of SBS-g-GOs network, and the original modification effect is even lower than that of the SBS itself.

### 3.4. Stability of SBS-g-GOs in Asphalt 

**Figure 10:**
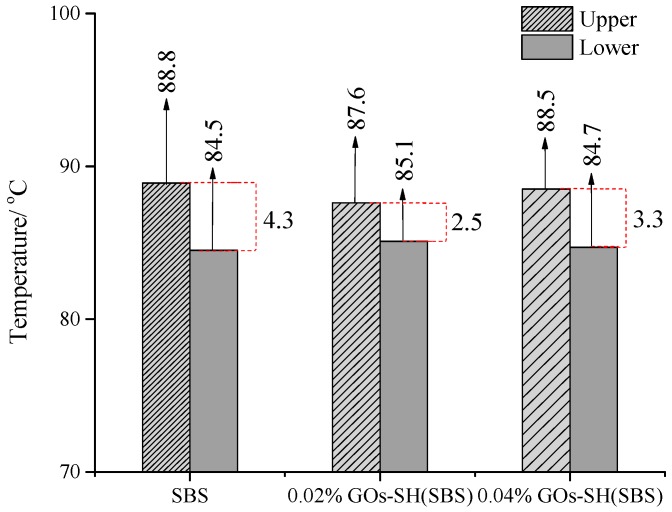
Test results of segregation experiment for various asphalt groups.

The degree of stability and uniform distribution of modifier in asphalt avoiding agglomeration greatly affect the performance of modified asphalt. Segregation experiment [47] was used to determine the dispersion degree of SBS-g-GOs in asphalt. In a typical test, asphalt was heated at 163 °C for 48 h and stabilized for 4 h, followed by quenching at −20 °C, followed by measurements of upper and lower layers for softening point. The difference in the softening points between the two layers for the same kind of asphalt represents the degree of segregation of modifiers in asphalt. The test results are shown in Figure 10.

Figure 10 suggests that after the isolation experiment, the upper and lower softening points of original SBS-modified asphalt were 88.8 °C and 84.5 °C, respectively, having a difference of 4.3 °C. On the contrary, 0.02% GOs-SH(SBS) and 0.04% GOs-SH(SBS) asphalt exhibited upper and lower softening pints of 87.6 °C, 85.1 °C and 88 °C, 84.7 °C, respectively. These results suggested that the degree of segregation of SBS in bitumen can be reduced by the addition of GOs-SH. Similar to DSR and MSCR results, 0.02% GOs-SH modified asphalt was observed with the best properties, while further additions of GOs-SH reacted with 1,4 C=C after the completion of 1,2 C=C reaction of SBS, thus destroying SBS’s own performance to varying degrees.

### 3.5. Microscopic Analysis

#### 3.5.1. Analysis of SBS-g-GOs in Asphalt via FM

**Figure 11:**
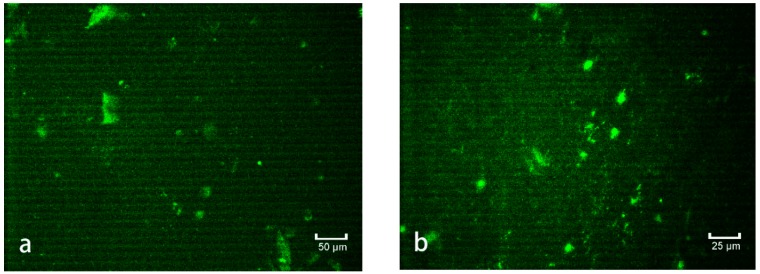

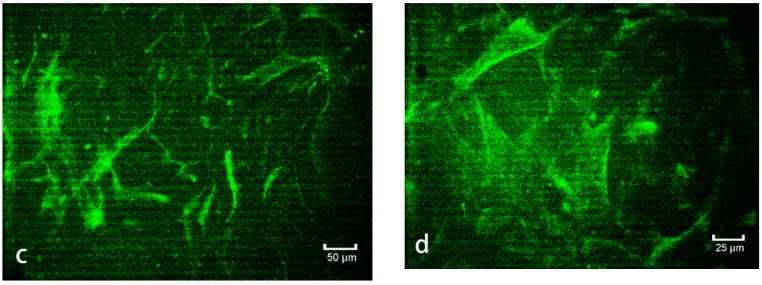
FM of SBS at 20 × 20 times (**a**) and 20 × 40 times (**b**), and SBS-g-GOs (0.04%) at 20 × 20 times (**c**) and 20 × 40 times (**d**).

For the dispersion of SBS in asphalt, FM studies were performed, and the results compiling the comparison of the newly formed SBS-g-GOs (0.04%) in asphalt before and after modification are shown in Figure 11a–d.

It can be clearly seen from Figure 11 that the undisturbed SBS has a relatively uniform dispersion in the asphalt but no obvious network structure. After the addition of GOs-SH, the newly formed SBS-g-GOs were uniformly dispersed in the asphalt with a clearly visible network structure. These observations superficially showed that GOs-SH can effectively promote the formation of SBS network structure.

#### 3.5.2. Microscopic Representation of GOs-SH

**Figure 12:**
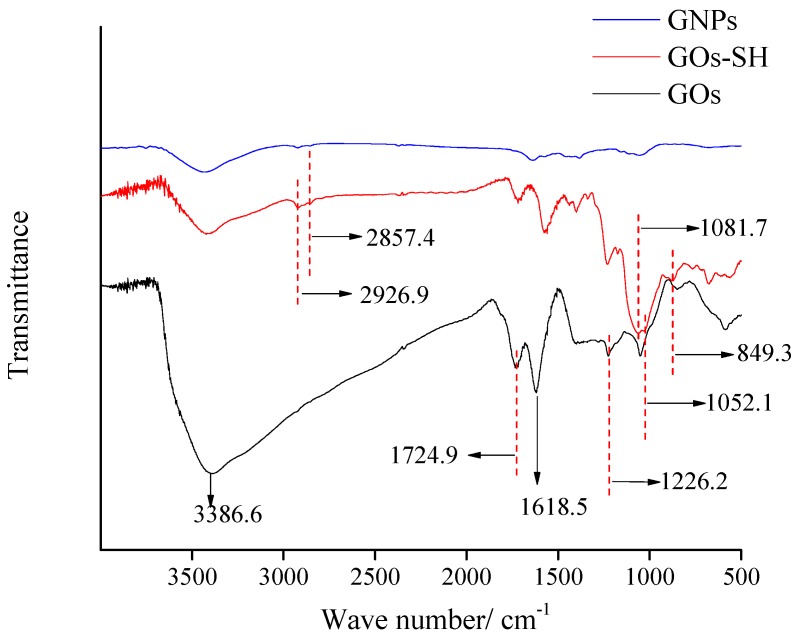
FT-IR spectra of GNPs, GOs-SH, and GOs.

Figure 12 shows the FT-IR spectra of GNPs, GOs-SH, and GOs. The absorption peaks at 3386.6 cm^−1^ and 1375.7 cm^−1^ represent the stretching and bending oscillation of the hydroxyl group (–OH), while that at 1724.90 cm^−1^ was ascribed to carboxyl group (–COOH) [53]. Similarly, the absorption peak at 1618.5 cm^−1^ was awarded to the characteristic absorption peak of GOs-adsorbed water [54]. The absorption peak at 1226.2 cm^−1^ and 1052.1 cm^−1^ were, respectively, ascribed to C–OH and C–O in C–O–C [55]. The weak absorption peak of mercapto group (–SH) in the grafted mercapto-silane coupling agent is weak and hence cannot be found in the spectrum of GOs-SH. However, the IR vibration peak of GOs at 3386 cm^−1^ was greatly weakened, indicating that most of the oxygen-containing functional groups on the graphene oxide surface reacted with the silane coupling agent, and obvious absorption peaks at 2926.9 cm^−1^ and 2857.1 cm^−1^ were observed [56]. The peak of the CH stretching vibration attributed to the methylene group on the mercaptosilane coupling agent, and the absorption peaks at 1081.7 cm^−1^ and 849.3 cm^−1^, were attributed to the anti-symmetric and symmetric stretching vibration peaks of Si–O–Si [56]. In brief, one can conclude from FT-IR results that the presence of characteristic peaks preliminarily confirmed the successful synthesis of GOs-SH.

**Figure 13:**
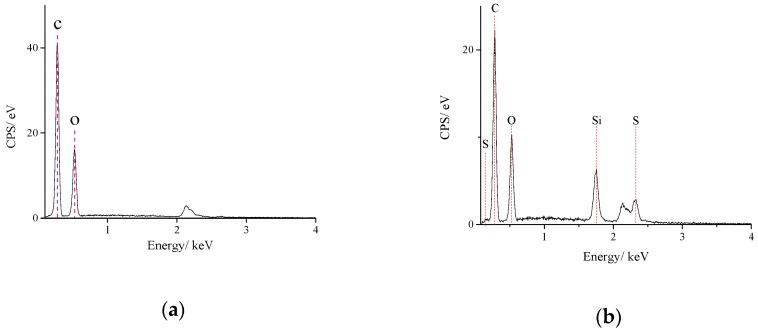
EDX analysis chart of GOs (**a**) and GOs-SH (**b**).

Figure 13a,b and Table S1 show the EDX analysis results of GOs and GOs-SH. It is obvious that the energy spectrum of S and Si appeared on the energy spectrum of GOs after modification, which is derived from –SH and –Si on the mercapto-silane coupling agent grafted on GOs during the reaction. These results also demonstrated the successful synthesis of GOs-SH.

**Figure 14:**
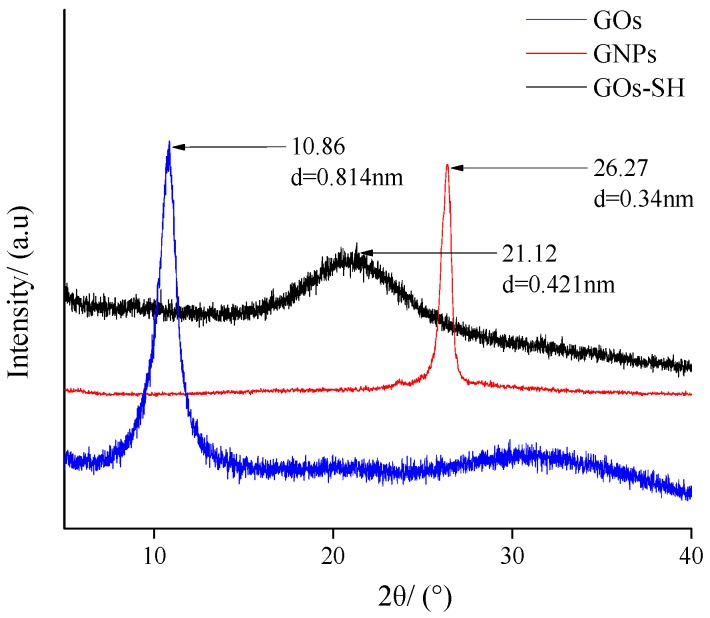
XRD patterns of GOs, GNPs, and GOs-SH.

Figure 14 shows the XRD patterns of GOs, GNPs, and GOs-SH. It is obvious that the GOs have a strong diffraction peak at 2*θ* = 10.86° [57] with an interplanar spacing of d = 0.814 nm calculated from Bragg’s formula [58]. GNPs showed strong diffraction peaks at 2*θ* = 26.27° having an interplanar spacing of d = 0.34 nm. However, the diffraction peak at 2*θ* = 21.12° of GOs exhibited an interplanar spacing between that of GOs and GNPs, i.e., d = 0.421 nm. The position of the diffraction peaks and the change in interplanar spacing indicated that GOs underwent a structural change after the reaction showing the formation of GOs-SH. From Figure 14, the diffraction peak of GOs-SH was shorter and wider compared to other samples. This is due to the diversity of the grafting sites of KH580 on GOs during the reaction providing multiple possibilities for GOs-SH. Compared with GOs, the interplanar spacing of GOs-SH reduced by about 48.3%, which could be due to the fact that part of KH580 is grafted and connected to different GO slices at the same time. Furthermore, due to the small size of KH580, GOs slices are compressed. Compared with GNPs, the interplanar spacing of GOs-SH has improved by about 23.82%, which is again due to the KH580 molecules interspersing with different degrees of grafting between the sheets during grafting process.

**Figure 15:**
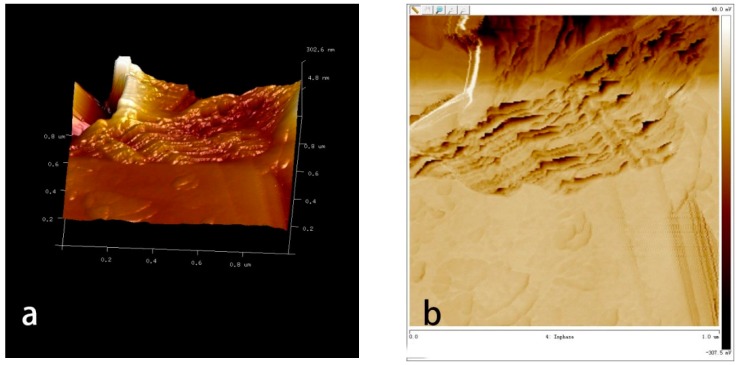

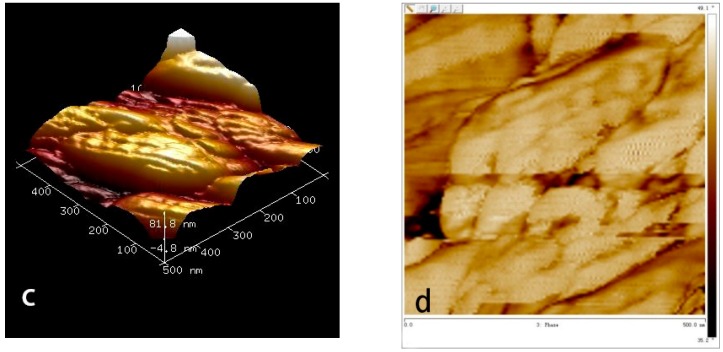
Appearance of GNPs (**a**,**b**) and GOs-SH (**c**,**d**) analyzed by AFM.

Figure 15 shows the AFM results of GNPs (a and b showing 3D height maps and phase diagrams, respectively) and GOs-SH (c and d). GNPs were observed with a dense multilayer sheet structure. Each piece of GNPs edge exhibits a certain degree of wavy undulations and is serrated at the cut. The overall surface of the lamina is smooth and flat, which was consistent with previous report [41]. Different from the morphology of GNPs, the 3D height map (c) and phase diagram (d) of GOs-SH suggest that the undulations of the sheet surface are increased after modification, while the edges of the sheet are thicker and smoother. At the same time, it can be clearly observed that GOs-SH has a small block-structure with fewer layers stacked. This is due to the fact that as single KH580 molecule can be grafted with up to three GOs, a larger amount of KH580 is required for the formation of a multilayer number of GOs-SH.

**Figure 16:**
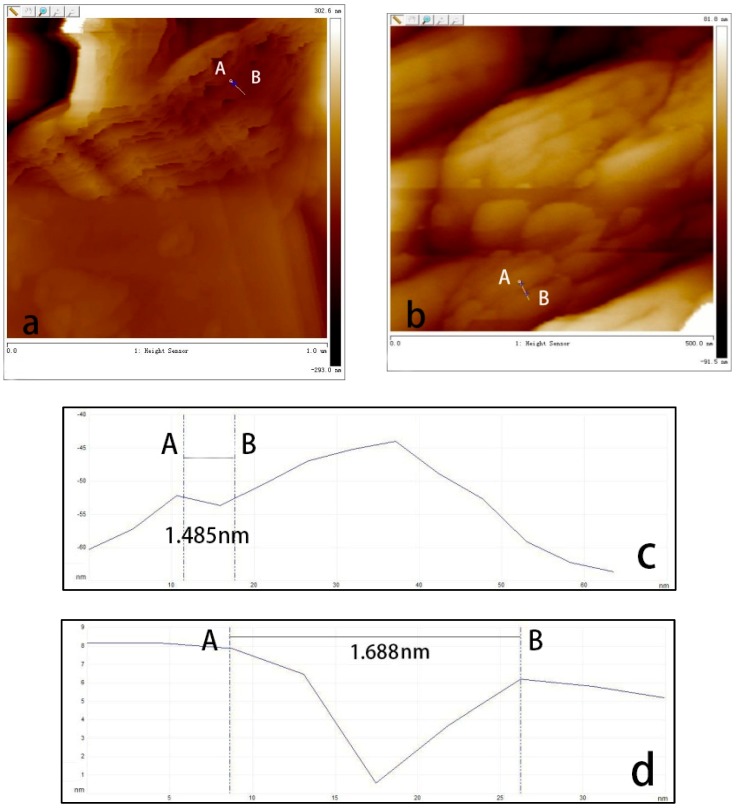
Single piece thickness test of GNPs (**a**,**c**) and GOs-SH (**b**,**d**) by AFM.

In order to measure the interlayer spacing, two adjacent sheets from the 2D height map of GNPs and GOs-SH were selected, as shown in Figure 16a,b. The interlayer spacing was 1.485 nm and 1.688 nm, respectively, as shown in Figure 16c,d. Combining the interplanar spacing results of GNPs (d = 0.34 nm) from the XRD analysis, the single piece thickness of GNP is approximately 1.145 nm, which is consistent with previous reports [59,60]. Following similar protocol, interplanar spacing of GOs-SH in the XRD analysis was d = 0.421 nm, while its calculated single piece thickness was about 1.267 nm. The thicker nature of GOs-SH compared to GNP was attributed to the grafting of its surface with KH580.

**Figure 17:**
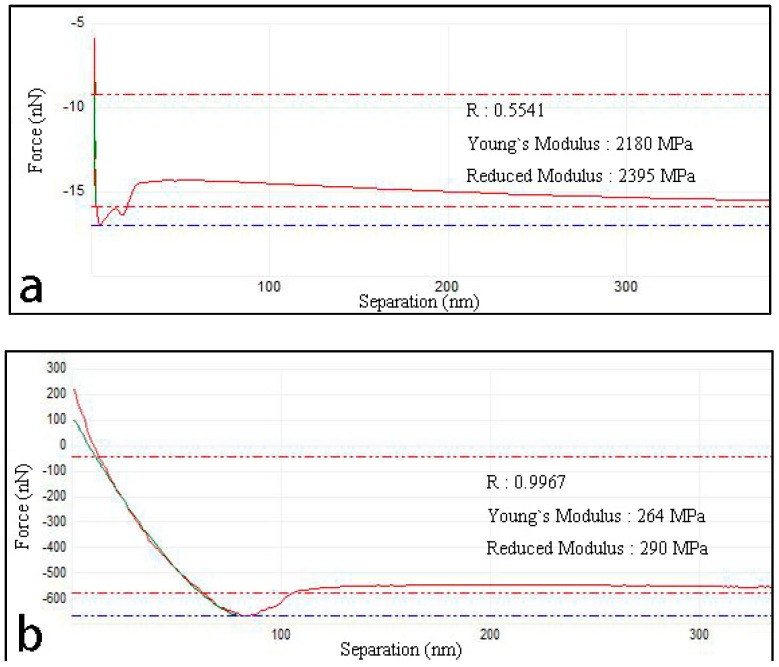
Mechanical properties analysis of GNPs (**a**) and GOs-SH (**b**) by AFM.

AFM can analyze the mechanical properties of material in a unique way, as shown in Figure 17. In AFM analysis, Young’s modulus is a theoretical physical quantity that describes the ability of a solid material to resist deformation, while reduced modulus is a physical value required to actually resist this deformation. From Figure 17a, it can be seen that the young’s modulus and reduced modulus of GOs-SH (264 MPa and 290 MPa) corresponded to a decrease of 87.895% compared to those of GNPs, i.e., 2180 MPa and 2395 MPa, respectively. These data suggested that GOs-SH had higher resistance to deformation than GNPs upon exposure to certain amount of pressure (attributed to higher bonding strength between base asphalt and GOs-SH molecules) than that between base asphalt and GNPs.

**Figure 18:**
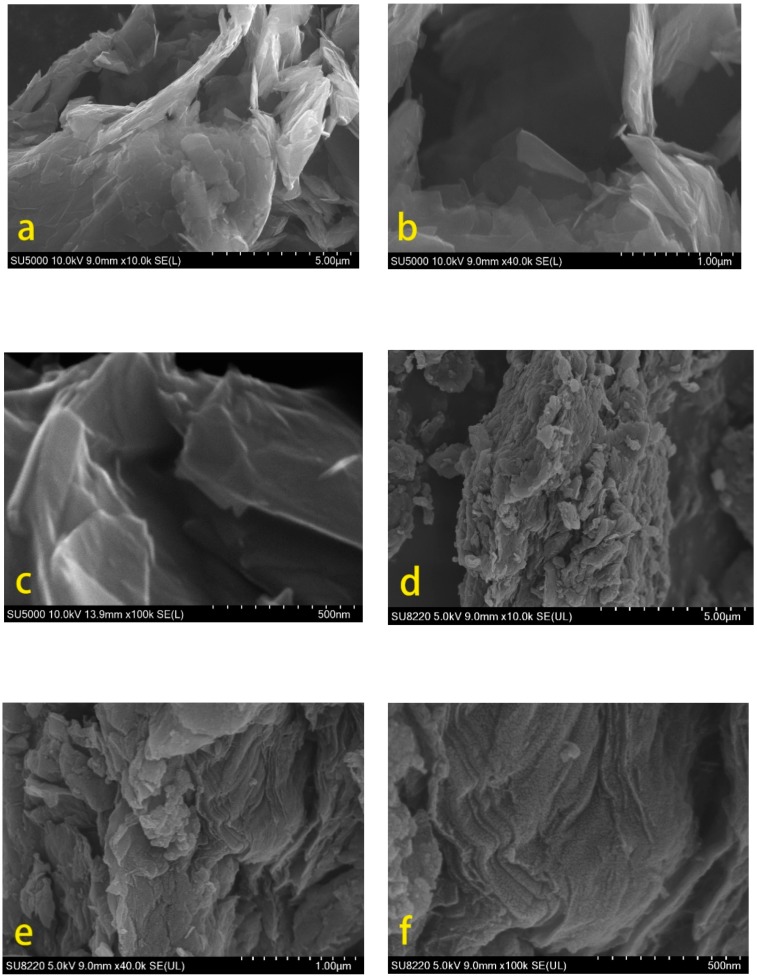
SEM analysis of GNPs (**a**–**c**) and GOs-SH (**d**–**f**).

SEM analyses were applied to analyze the appearance of GNPs and GOs-SH, and the images are displayed in Figure 18. Figure 18a–c suggests that GNPs have a clear sheet structure with smooth surface. The edge of the sheet possessed few wrinkles with low thickness. These facts are consistent with the morphology of GNPs as obtained by AFM analysis in Figure 15a,b. Figure 18d–f shows thicker slices of GOs-SH with increased degree of undulation than GNPs. There also appeared wavy bends at the sheet edge with variable degrees of curling. This change in morphology is caused by the grafting reaction on the surface and edge of GOs. These characteristics of GOs-SH collected from SEM results were consistent with those obtained via AFM analysis in Figure 15c,d.

**Figure 19:**
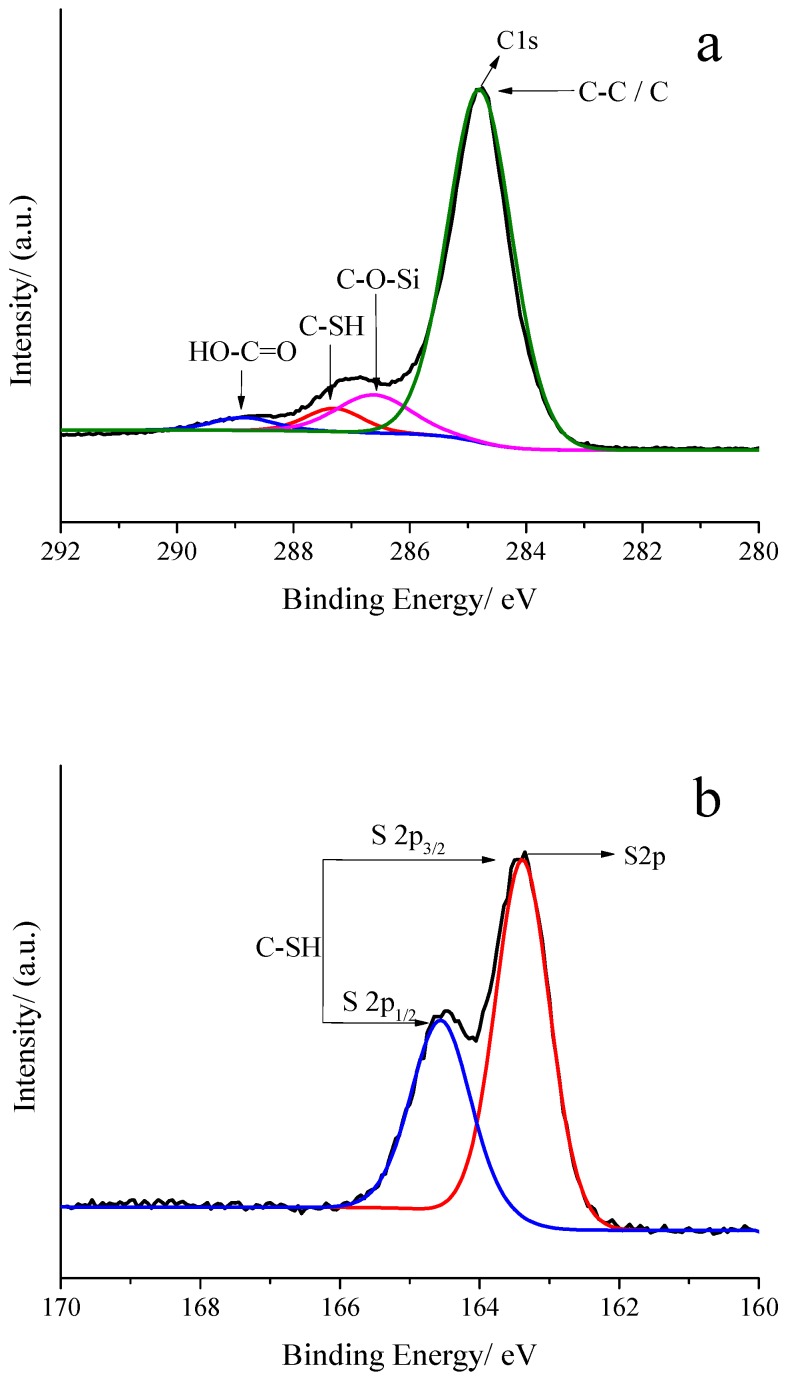
High resolution XPS spectra of GOs-SH of C1s (**a**) and S2p (**b**).

Figure 19 and Table S2 compile the XPS analyses results about the elemental composition and electronic states of the elements on the GOs-SH. XPS spectrum of C1s in Fig 19a can be split into four peaks. The strongest peak (82.2% of the total peak area) at 284.79 eV binding energy represents C–C or elemental C in GOs [61], while the one at 286.61 eV was ascribed to C–O–Si [62]. Another peak at 287.28 eV represents C–SH (from KH580) [61,62], while a very small peak at 288.86 eV was awarded to HO–C=O [61,63]. The presence of C–O–Si peak confirmed the successful grafting onto GOs. The peak-to-area ratio of C–SH to C–O–Si was 3.56:1, which is close to the ratio of three C–O–Si bonds produced by the grafting of one –SH, as shown in the reaction mechanism in Figure 2, confirming the proposed route for the reaction. A small amount of HO–C=O peak indicated the occurrence of mild oxidation on the GOs after the reaction.

Figure 19b shows the high resolution S2p XPS spectrum of GOs-SH, which can be divided into S2p_1/2_ and S2p_3/2_ due to spin-orbit coupling at 164.55 eV and 163.39 eV, confirming the existence of sulfhydryl group with a combined energy difference of 1.16 eV [63,64]. 

**Figure 20:**
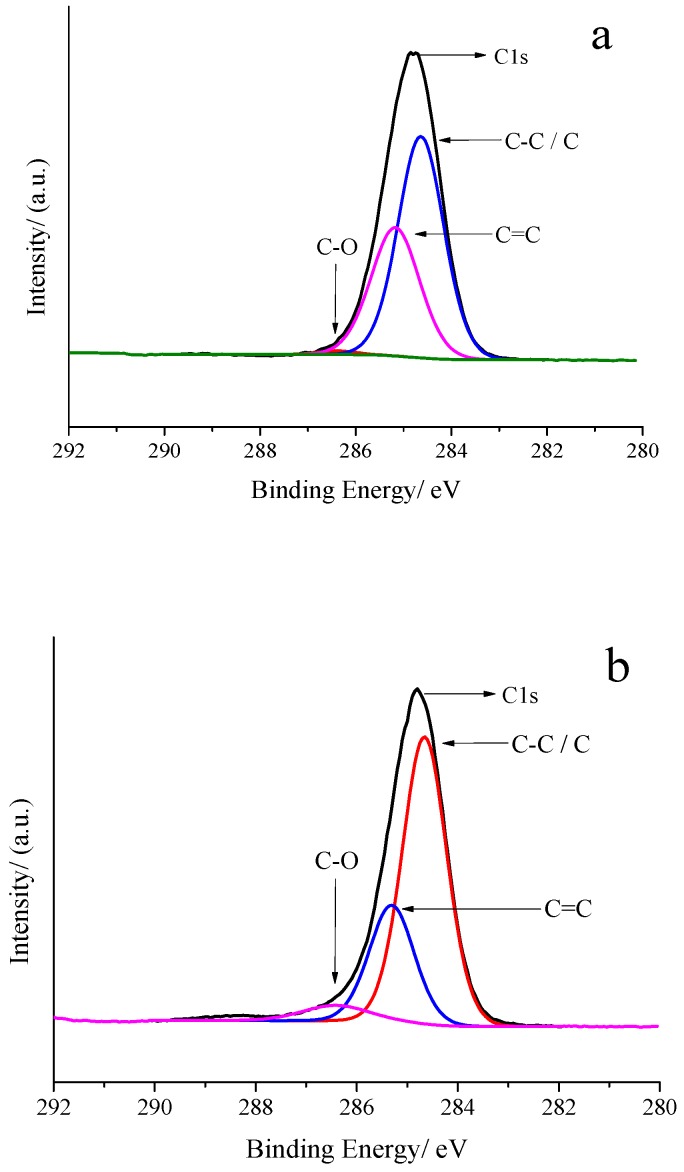
XPS spectra of C1s of original SBS-modified asphalt (**a**) and SBS-g-GOs (0.04% GOs-SH) modified asphalt (**b**).

The C1s spectrum of original SBS-modified asphalt in Figure 20a can be fitted into three peaks, i.e., the one at 284.64 eV represents C–C, while that at 285.28 eV represents C=C [65]. The smallest peak at 286.38 eV represents C–O. The peak area ratio of C–C to C=C was nearly 1:1.614, in which the C=C bond was derived from the aromatics in asphalt, which is same as C–O and 1, 4 vinyl and 1, 2 vinyl of SBS. After grafting onto GOs-SH (0.04%), the C1s spectrum of SBS-g-GOs-modified asphalt shown in Figure 20b can be divided into three peaks. The peak at 284.66 eV represents C–C or elemental C (GOs); at 285.31 eV it was ascribed to C=C, while the one at 286.32 eV represents C–O. A decrease and increase in the peak area of C=C bond and C–C bond, respectively, compared to that of SBS (Table S2) indicates that a portion of the C=C bond is converted into a C–C bond, by the reaction of GOs-SH with the vinyl group in SBS. The unsaturated, derived nature of most C=C bonds in asphalt would not favor thiol-ene click reaction, thus facilitating GOs-SH to undergo a thiol-ene click reaction with SBS in asphalt. The increase in the C–O peak area is due to the presence of certain C–O and reacted HO–OC=O functional groups in GOs-SH, as shown by the reaction mechanism in Figure 2. No peak of C–S appeared in the C1s XPS spectrum, presumably due to its low content or overlapping by other functional groups in its vicinity. However, S-containing functional groups in the two kinds of asphalts were observed in XPS spectra in Figure S3.

S2p spectra of original SBS-modified asphalt (Figure S3a) showed C–S or elemental S (stabilizer) peak at 163.86 eV and C–SO–C peak at 165.16 eV [62,66,67]. The minimum peak at 169.02 eV represents (SO_4_)^2−^. The peak of C–SO–C and SO_4_^2−^ could result from S radicals due to high temperature stabilizer [68,69,70]. S2p spectrum of SBS-g-GOs-modified asphalt (Figure S3b) suggested similar species with an increase in C–S bond peak area (Table S3) compared to that of pristine SBS, which could be due to thiol-ene click reaction of sulfhydryl group on GOs-SH with C=C bond in asphalt.

## 4. Conclusions

In summary, this study reported the preparation of SBS-g-GOs-modified asphalt by grafting thiolated GOs (GOs-SH) with SBS using the thiol-ene click reaction in an asphalt environment. The results of three indicators and the DSR test demonstrated that an appropriate amount of GOs-SH can effectively improve the plasticity, low-temperature performance, and viscoelastic properties of SBS-modified asphalt without changing the PG grade. MSCR test and FM results demonstrated enhanced the high-temperature anti-rutting performance of GOs-SH-added SBS-g-GOs asphalt compared with original SBS-modified asphalt and GNPs/SBS-modified asphalt attributed to the promotion and formation of SBS network structure by GO-SH. SBS-g-GOs were confirmed to be highly stable and evenly dispersed in asphalt matrix. The thiolization of GOs was confirmed by FTIR and EDX analysis. AFM results proved that thiolated GOs have greater elasticity than GNPs. Qualitative and quantitative analyses of functional groups in GOs-SH, SBS-modified asphalt and SBS-g-SH modified asphalt were confirmed by XPS analyses, emphasizing the realization of the thiol-ene click reaction between GOs-SH and SBS in asphalt. The current study, which is based on the application of click chemistry for the preparation of GOs-modified SBS based asphalt, can be deemed a promising approach for industrial applications.

## Figures and Tables

**Table 1:** RF (kPa) of each sample as a function of temperature.

*θ*/°C	SBS	0.05% GNPs/SBS	0.02% GOs-SH (SBS)	0.04% GOs-SH (SBS)	0.06% GOs-SH (SBS)
46	49.55	54.86	70.04	62.34	51.33
52	26.21	28.52	34.43	29.86	26.21
58	15.16	16.01	18.07	15.00	13.96
64	9.35	8.75	10.14	8.17	7.52
70	5.79	5.06	5.96	4.66	4.18
76	3.66	3.50	3.59	2.73	2.47
82	2.41	2.24	2.19	1.65	1.60
**RTFOT**					
76	-	-	-	-	2.88
82	2.74	2.46	2.41	2.58	1.97
PG	82	82	82	82	76

**Table 2:** R and J_nr_ of various samples at 0.1 KPa and 3.2 KPa.

Type/Unit	SBS	0.05% GNPs/SBS	0.02% GOs-SH (SBS)	0.04% GOs-SH (SBS)
R_0.1_	0.675	0.686	0.786	0.745
R_3.2_	0.341	0.368	0.494	0.417
J_nr0.1_/ kPa^−1^	0.856	0.826	0.553	0.660
J_nr3.2_/ kPa^−1^	2.655	2.158	1.618	1.882

## Supplementary Materials

The following are available online at http://www.mdpi.com/2073-4360/10/11/1264/s1, Figure S1: 3D simulation of GOs-SH macromolecules, Figure S2: Schematic representation of preparation process of modified asphalt, Figure S3: S2p XPS spectra of original SBS modified asphalt (a) and SBS-g-GOs (0.04% GOs-SH) modified asphalt (b), Table S1: EDX element distribution of GNPs and GOs-SH, Table S2: The composition, and electronic states of the elements (C1s and S2p) about the GOs-SH, Table S3: The composition, and electronic states of the elements (C1s and S2p) on the surface of synthesized modified asphalt (SBS and SBS-g-GOs).

## References

[1] Wang P., Dong Z.J., Tan Y.Q., Liu Z.Y. (2017). Effect of multi-walled carbon nanotubes on the performance of styrene–butadiene–styrene copolymer modified asphalt. Mater. Struct..

[2] Chen J., Wang H., Li M., Li L. (2016). Evaluation of pavement responses and performance with thermal modified asphalt mixture. Mater. Des..

[3] Baek J., Sang Y.L., Lee H.J. (2018). Comparative evaluation of wma additives effects on conventional and polymer modified asphalt pavements. KSCE J. Civ. Eng..

[4] Liang M., Xin X., Fan W., Ren S., Shi J., Luo H. (2017). Thermo-stability and aging performance of modified asphalt with crumb rubber activated by microwave and TOR. Mater. Des..

[5] Santagata E., Baglieri O., Dalmazzo D., Tsantilis L. (2013). Evaluation of the anti-rutting potential of polymer-modified binders by means of creep-recovery shear tests. Mater. Struct..

[6] Dong F., Zhao W., Zhang Y., Wei J., Fan W., Yu Y., Wang Z. (2014). Influence of SBS and asphalt on SBS dispersion and the performance of modified asphalt. Constr. Build. Mater..

[7] Hou D., Han M., Muhammad Y., Liu Y., Zhang F., Yin Y., Duan S., Li J. (2018). Performance evaluation of modified asphalt based trackless tack coat materials. Constr. Build. Mater..

[8] Goli A., Ziari H., Amini A. (2017). Influence of carbon nanotubes on performance properties and storage stability of SBS modified asphalt binders. J. Mater. Civ. Eng..

[9] Pang J., Du S., Chang R., Cui D. (2016). Rheological properties of SBS-modified asphalt in the presence of dithiodimorpholine and tetraethyl thiuram disulfide. Polym. Compos..

[10] Sengul C.E., Oruc S., Iskender E., Aksoy A. (2013). Evaluation of SBS modified stone mastic asphalt pavement performance. Constr. Build. Mater..

[11] Wu G., Zeng S., Ou E., Yu P., Lu Y., Xu W. (2010). Photoinitiator grafted styrene–butadiene–styrene triblock copolymer. Mater. Sci. Eng. C.

[12] Cong P., Chen S., Chen H. (2011). Preparation and properties of bitumen modified with the maleic anhydride grafted styrene-butadiene-styrene triblock copolymer. Polym. Eng. Sci..

[13] Kennedy J.E., Lyons J.G., Geever L.M., Higginbotham C.L. (2009). Synthesis and characterisation of styrene butadiene styrene-g-acrylic acid for potential use in biomedical applications. Mater. Sci. Eng. C.

[14] Mitov Z., Velichkova R. (1993). Modification of styrene-isoprene block copolymers—3. Addition of maleic anhydride—Mechanism. Eur. Polym. J..

[15] Hsiue G.H., Huang W.K., Hou W.H. (1989). Dynamic mechanical and dielectric properties of epoxidized SBS triblock copolymer. J. Polym. Sci. Part A Polym. Chem..

[16] Zhang A., Li C. (2003). Chemical initiation mechanism of maleic anhydride grafted onto styrene–butadiene–styrene block copolymer. Eur. Polym. J..

[17] Decker C., Nguyen Thi Viet T. (2015). High-speed photocrosslinking of thermoplastic styrene–butadiene elastomers. J. Appl. Polym. Sci..

[18] Schapman F., Couvercelle J.P., Bunel C. (2000). Low molar mass polybutadiene made crosslinkable by silane moities introduced via addition of thiol to double bond: 4. Crosslinking study. Polymer.

[19] Lotti L., Coiai S., Ciardelli F., Galimberti M., Passaglia E. (2010). Thiol-ene radical addition of l-cysteine derivatives to low molecular weight polybutadiene. Macromol. Chem. Phys..

[20] Li X.K., Chen G.S., Duan M.W., Yang W.C., Tang S.C., Cao Y.D., Luo Y., Li X.K., Chen G.S., Duan M.W. (2017). Branched hydroxyl modification of SBS using Thiol-ENE reaction and its subsequent application in modified asphalt. Ind. Eng. Chem. Res..

[21] Xi W., Scott T.F., Kloxin C.J., Bowman C.N. (2014). Click chemistry in materials science. Adv. Funct. Mater..

[22] Guo J., Xie Z., Tran R.T., Xie D., Yang J. (2014). Click chemistry plays a dual role in biodegradable polymer design. Adv. Mater..

[23] Lodge T.P. (2015). A virtual issue of macromolecules: “Click chemistry in macromolecular science”. Macromolecules.

[24] Logan A., Pell V.R., Shaffer K.J., Evans C., Stanley N.J., Robb E.L., Prime T.A., Chouchani E.T., Cochemé H.M., Fearnley I.M. (2016). Assessing the mitochondrial membrane potential in cells and in vivo using targeted click chemistry and mass spectrometry. Cell MeTable.

[25] Döhler D., Michael P., Binder W.H. (2017). CuAAC-based click chemistry in self-healing polymers. Acc. Chem. Res..

[26] Bian T., Wang C., Lu Z., Xie R., Yang Q.Z., Wu L.Z., Tung C.H., Liu Z., Yin Y., Zhang T. (2015). Nanocrystals: A versatile ‘click chemistry’ route to size-restricted, robust, and functionalizable hydrophilic nanocrystals (small 14/2015). Small.

[27] Elchinger P.H., Faugeras P.A., Boens B., Brouillette F.O., Montplaisir D., Zerrouki R., Lucas R. (2011). Polysaccharides: The “click” chemistry impact. Polymers.

[28] Fouassier J.P., Rabek J.F. (1993). Radiation Curing in Polymer Science and Technology.

[29] Hoyle C.E. (2010). Thiol–enes: Chemistry of the past with promise for the future. J. Polym. Sci. Part A Polym. Chem..

[30] Antoni P., Robb M.J., Campos L., Montanez M., Hult A., Malmström E., Malkoch M., Hawker C.J. (2010). Pushing the limits for thiol−ene and CuAAC reactions: Synthesis of a 6th generation dendrimer in a single day. Macromolecules.

[31] Montañez M.I., Campos L.M., Antoni P., Hed Y., Walter M.V., Krull B.T., Khan A., Hult A., Hawker C.J., Malkoch M. (2010). Accelerated growth of dendrimers via thiol−ene and esterification reactions. Macromolecules.

[32] Lim Y., Heo G.S., Rezenom Y.H., Pollack S., Raymond J.E., Elsabahy M., Wooley K.L. (2014). Development of a vinyl ether-functionalized polyphosphoesteras a template for multiple postpolymerization conjugation chemistriesand study of core degradable polymeric nanoparticles. Macromolecules.

[33] Ma J., Bartels J.W., Li Z., Zhang K., Cheng C., Wooley K.L. (2010). Synthesis and solution-state assembly or bulk state thiol-ene crosslinking of pyrrolidinone- and alkene-functionalized amphiphilic block fluorocopolymers: From functional nanoparticles to anti-fouling coatings. Aust. J. Chem..

[34] Ma J., Cheng C., Wooley K.L. (2009). The power of raft for creating polymers having imbedded side-chain functionalities: Norbornenyl-functionalized polymers and their transformations via romp and thiol-ene reactions. Aust. J. Chem..

[35] Li Z., Guo Q., Li Z., Fan G., Xiong D., Su Y., Zhang J., Zhang D. (2015). Enhanced mechanical properties of graphene (reduced graphene oxide)/aluminum composites with a bioinspired nanolaminated structure. Nano Lett..

[36] Guardia L., Villar-Rodil S., Paredes J.I., Rozada R., Martínez-Alonso A., Tascón J. (2012). UV light exposure of aqueous graphene oxide suspensions to promote their direct reduction, formation of graphene–metal nanoparticle hybrids and dye degradation. Carbon.

[37] Shen J., Hu Y., Li C., Qin C., Ye M. (2010). Synthesis of amphiphilic graphene nanoplatelets. Small.

[38] Li R., Xiao F., Amirkhanian S., You Z., Huang J. (2017). Developments of nano materials and technologies on asphalt materials—A review. Constr. Build. Mater..

[39] Gao C.M., Han S., Chen S., Li H. (2014). Research on basalt fiber asphalt concrete’s low temperature performance. Appl. Mech. Mater..

[40] Le J., Marasteanu M., Turos M. (2016). Graphene Nanoplatelet (GNP) Reinforced Asphalt Mixtures: A Novel Multifunctional Pavement Material.

[41] Han M., Li J., Muhamma Y., Hou D., Zhang F., Yin Y., Duan S. (2018). Effect of polystyrene grafted graphene nanoplatelets on the physical and chemical properties of asphalt binder. Constr. Build. Mater..

[42] Han M., Li J., Muhammad Y., Yin Y., Yang J., Yang S., Duan S. (2018). Studies on the secondary modification of SBS modified asphalt by the application of octadecyl amine grafted graphene nanoplatelets as modifier. Diam. Relat. Mater..

[43] Li J., Han M., Muhammad Y., Liu Y., Yang S., Duan S., Huang W., Zhao Z. (2018). Comparative analysis, road performance and mechanism of modification of polystyrene graphene nanoplatelets (PS-GNPS) and octadecyl amine graphene nanoplatelets (ODA-GNPS) modified SBS incorporated asphalt binders. Constr. Build. Mater..

[44] Airey G.D. (2004). Styrene butadiene styrene polymer modification of road bitumens. J. Mater. Sci..

[45] Zhang F., Li J., Yaseen M., Han M., Yin Y., Yang S. (2018). Preparation methods and performance of modified asphalt using rubber–plastic alloy and its compounds. J. Mater. Civ. Eng..

[46] Zhang F., Muhammad Y., Liu Y., Han M., Yin Y., Hou D., Li J. (2018). Measurement of water resistance of asphalt based on surface free energy analysis using stripping work between asphalt-aggregate system. Constr. Build. Mater..

[47] Ministry of Transport of the People’s Republic of China (2011). Standard Test Methods of Bitumen and Bituminous Mixtures for Highway Engineering: JTG E20-2011.

[48] Jahanbakhsh H., Karimi M.M., Nejad F.M., Jahangiri B. (2016). Viscoelastic-based approach to evaluate low temperature performance of asphalt binders. Constr. Build. Mater..

[49] Yang X., You Z. (2015). High temperature performance evaluation of bio-oil modified asphalt binders using the DSR and MSCR tests. Constr. Build. Mater..

[50] Huang W., Tang N. (2015). Characterizing SBS modified asphalt with sulfur using multiple stress creep recovery test. Constr. Build. Mater..

[51] Shaheen M., Al-Mayah A., Tighe S. (2016). Optimization of hot-mix asphalt surface course mix design for fatigue resistance: High-friction aggregate and PG plus. J. Mater. Civ. Eng..

[52] Norouzi A., Kim Y.R., Kim S.S., Yang J. (2017). Effect of reclaimed asphalt pavement content and binder grade on fatigue-resisting performance of asphalt mixtures in Georgia. J. Mater. Civ. Eng..

[53] Wen T., Wu X., Tan X., Wang X., Xu A. (2013). One-pot synthesis of water-swellable mg-al layered double hydroxides and graphene oxide nanocomposites for efficient removal of As(V) from aqueous solutions. ACS Appl. Mater. Interfaces.

[54] Stankovich S., Piner R.D., Nguyen S.T., Ruoff R.S. (2006). Synthesis and exfoliation of isocyanate-treated graphene oxide nanoplatelets. Carbon.

[55] Ma Z., Qiu Y., Yang H., Huang Y., Liu J., Lu Y., Zhang C., Hu P. (2015). Effective synergistic effect of dipeptide-polyoxometalate-graphene oxide ternary hybrid materials on peroxidase-like mimics with enhanced performance. ACS Appl. Mater. Interfaces.

[56] Li X., Chen G., Yang W., Duan M., Tang S., Cao Y., Mai Y., Luo Y. (2017). Carboxyl branched-functionalized modification of SBS and application to modified asphalt. Eng. Plast. Appl..

[57] Li S.M., Wang Y.S., Hsiao S.T., Liao W.H., Lin C.W., Yang S.Y., Tien H.W., Ma C.C.M., Hu C.C. (2015). Fabrication of a silver nanowire-reduced graphene oxide-based electrochemical biosensor and its enhanced sensitivity in the simultaneous determination of ascorbic acid, dopamine, and uric acid. J. Mater. Chem. C.

[58] Zhu C., Zhai J., Wen D., Dong S. (2012). Graphene oxide/polypyrrole nanocomposites: One-step electrochemical doping, coating and synergistic effect for energy storage. J. Mater. Chem..

[59] Ren L., Wang X., Guo S., Liu T. (2011). Functionalization of thermally reduced graphene by in situ atom transfer radical polymerization. J. Nanopart. Res..

[60] Murdock A.T., Koos A., Britton T.B., Houben L., Batten T., Zhang T., Wilkinson A.J., Duninborkowski R.E., Lekka C.E., Grobert N. (2013). Controlling the orientation, edge geometry, and thickness of chemical vapor deposition graphene. ACS Nano.

[61] Vogt A.D., Taejoon Han A., Beebe T.P. (1997). Adsorption of 11-mercaptoundecanoic acid on ni(111) and its interaction with probe molecules. Langmuir.

[62] Beamson G., Briggs D. (1992). High Resolution XPS of Organic Polymers: Scienta ESCA300 Database.

[63] Duan W., Li M., Sun H., Cheng J., Zhang J. (2017). Preparation and characterization of thiol-ene/epoxy photo-thermal double curing system. Bonding.

[64] Andreu N., Flahaut D., Dedryvere R., Minvielle M., Martinez H., Gonbeau D. (2015). XPS investigation of surface reactivity of electrode materials: Effect of the transition metal. ACS Appl. Mater. Interfaces.

[65] Hai G.H., Ying H.C., Jing Y.H., Hua Tang H., Guo Q.X. (2005). Formation of a tetra-σ-bonded intermediate in acetylethyne binding on si(100)-2 × 1. Langmuir.

[66] Luckas N., Gotterbarm K., Streber R., Lorenz M.P., Höfert O., Viñes F., Papp C., Görling A., Steinrück H.P. (2011). Adsorption and reaction of SO_2_ on clean and oxygen precovered Pd(100)-a combined HR-XPS and DF study. Phys. Chem. Chem. Phys..

[67] Dalili N., He A., Liu Q., Ivey D.G. (2010). Erratum to: A cryo-xps study of triammonium citrate-kaucl_4_-na_2_so_3_ electroplating solutions for pb-free solder packaging. J. Electron. Mater..

[68] Yang X., Liu K. (2008). Study on mechanism of sulphur modified asphalt. J. Shijiazhuang Railw. Inst..

[69] Yang X., Xiong S., Jiao S., Liu K. (2009). Investigation on the properties of sulphur modified asphalt mixture and it’s modifying mechanism. J. Hunan Univ. Sci. Technol..

[70] Iii S.B.C., Mohammad L.N., Elseifi M.A. (2011). Laboratory performance characteristics of sulfur-modified warm-mix asphalt. J. Mater. Civ. Eng..

