# Supplementary material for: Preparation and Properties of SBS-g-GOs-Modified Asphalt Based on a Thiol-ene Click Reaction in a Bituminous Environment
Source: Polymers (Basel). 2018 Nov 13;10(11):1264. doi: 10.3390/polym10111264 (PMC6401793; doi:10.3390/polym10111264)
Supplement: Supplementary file 1 [file polymers-10-01264-s001.pdf]

Supplementary Materials: Preparation and Properties of SBS-g-GOs Modified Asphalt Based on Thiol-ene Click Reaction in a Bituminous Environment

Jing Li, Meizhao Han, Yaseen Muhammad, Yu Liu, Zhibin Su, Jing Yang, Song Yang and Shaochan Duan

Table S1. EDX element distribution of GNPs and GOs-SH.

Type	C/%	Si/%	O/%	S/%	N/%
GNPs	60.21	0	39.06	0	0.73
GOs-SH	45.90	22.27	20.80	10.81	0.21

Table S2. The composition, and electronic states of the elements (C1s and S2p) about the GOs-SH.

Name	Position/eV	Area/%
	**Spectrum of C1s**	
C-C / C	284.79	82.2
C-O-Si	286.61	10.17
C-SH	287.28	2.86
HO-C=O	288.86	2.16
	**Spectrum of S2p**	
S2p (3/2)	163.39	63.37
S2p (1/2)	164.55	36.63

Table S3. The composition, and electronic states of the elements (C1s and S2p) on the surface of synthesized modified asphalt (SBS and SBS-g-GOs).

Name	Position/eV		Area/%	
	SBS	SBS-g-GOs	SBS	SBS-g-GOs
		**Spectrum of C1s**		
C-C / C	284.64	284.66	61.26	67.06
C=C	285.28	285.31	37.95	27.64
C-O	286.38	286.32	0.79	5.3
		**Spectrum of S2p**		
C-S / S	163.86	163.93	59.49	78.4
C-SO-C	165.16	165.27	26.72	8.88
SO4(-2)	169.02	169.12	13.79	12.72

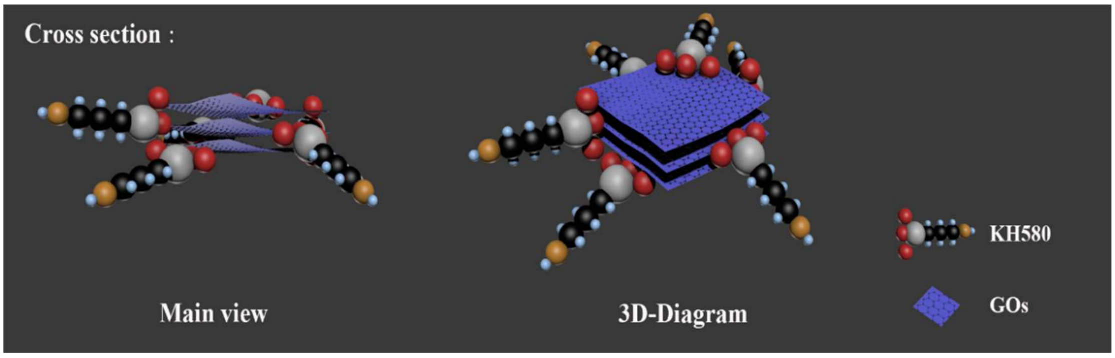

Figure S1. 3D simulation of GOs-SH macromolecules.

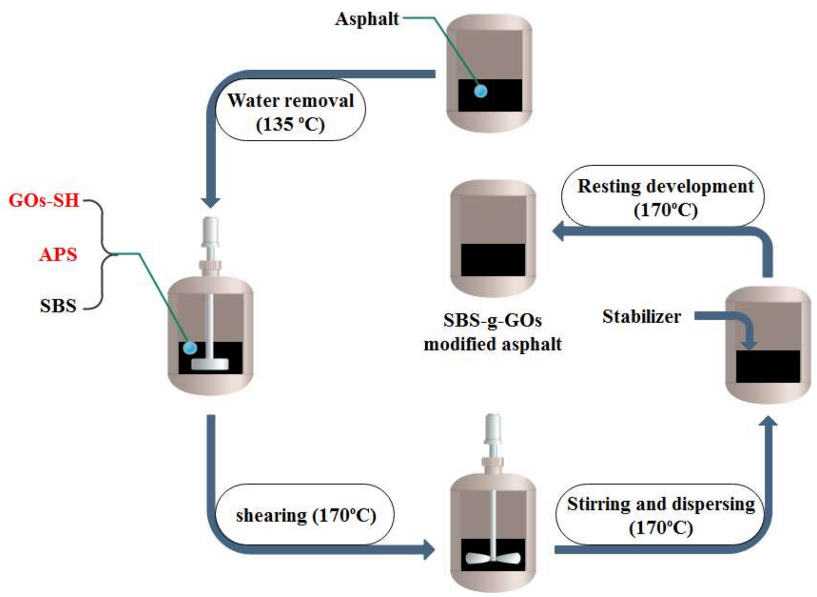

Figure S2. Schematic representation of preparation process of modified asphalt.

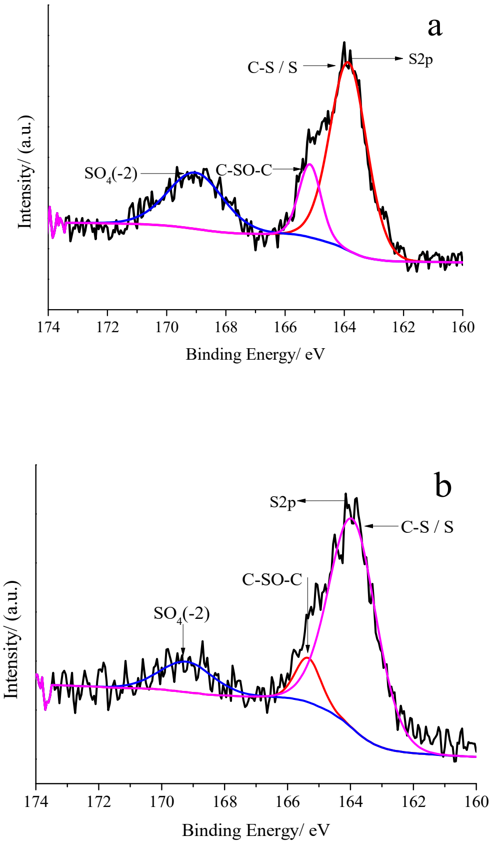

Figure S3. S2p XPS spectra of original SBS modified asphalt (**a**) and SBS-g-GOs (0.04% GOs-SH) modified asphalt (**b**).
